# A Case of Bilateral Vertebral Artery Dissecting Aneurysm Treated With Multimodality Therapy Under Superficial Temporal Artery Assistance–Posterior Cerebral Artery Bypass

**DOI:** 10.7759/cureus.45326

**Published:** 2023-09-15

**Authors:** Yusuke Sakamoto, Ryusuke Kabeya, Masahiro Nishihori

**Affiliations:** 1 Department of Neurosurgery, Japanese Red Cross Aichi Medical Center Nagoya Daini Hospital, Nagoya, JPN; 2 Department of Neurosurgery, Ichinomiya Municipal Hospital, Ichinomiya, JPN; 3 Department of Neurosurgery, Nagoya University Graduate School of Medicine, Nagoya, JPN

**Keywords:** insurance bypass, multimodality therapy, bilateral vertebral artery dissecting aneurysm, superficial temporal artery-posterior cerebral artery bypass, superficial temporal artery-superior cerebellar artery bypass

## Abstract

A ruptured bilateral vertebral artery dissecting aneurysm (BVDA) is a challenging vascular disorder. Trapping surgery with bypass assistance could be a potential treatment; however, there is a risk of ischemic complications. Recently, endovascular treatment has been reported, but its long-term outcomes remain uncertain.

The patient was a 57-year-old male who presented with subarachnoid hemorrhage. Digital subtraction angiography showed a dilated dominant left vertebral artery (VA) and a narrowed right VA, suggesting a BVDA. First, we performed a right superficial temporal artery-superior cerebellar artery (STA-SCA) insurance bypass. We then performed proximal clipping of the left vertebral VA. The pulsation of the STA-SCA bypass disappeared on day 6. Three-dimensional computed tomography angiography (3DCTA) showed the emergence of a fusiform aneurysm and proximal stenosis of the contralateral VA. On day 31, we performed a superficial temporal artery-posterior cerebral artery (STA-PCA) insurance bypass. Stent-assisted coil embolization was planned for two days after the STA-PCA bypass. However, preoperative angiography showed progression of right proximal VA stenosis, and stenting appeared impossible. There was no change in somatosensory evoked potential (SEP), and angiography showed sufficient retrograde blood flow to the posterior circulation during the right VA balloon occlusion test (BOT). Therefore, internal trapping of the right VA was performed. Postoperative angiography showed perfect patency of the left STA-PCA bypass and retrograde blood flow to the posterior circulation. There was no additional neurological deficit after endovascular treatment.

Multimodality therapy could be a potential treatment for bilateral VA dissection.

## Introduction

A ruptured bilateral vertebral artery dissecting aneurysm (BVDA) is a challenging and lethal vascular disorder [1]. Simple trapping of the unilateral vertebral artery (VA) sometimes leads to enlargement of the contralateral aneurysm because of the increase in hemodynamic stress [2-4]. Trapping on the bilateral VA as a point of hemostasis is a radical treatment that may result in fatal ischemic complications of the brainstem or posterior circulation. Trapping surgery with bypass assistance could be one potential treatment option, but there is an unpredictable risk of ischemic complications related to thrombosis of the perforator around the vertebrobasilar stump lesion [5]. Moreover, bypass to the posterior circulation requires advanced microsurgical techniques. Recently, endovascular treatments, including flow-diverting devices, have been reported [6-8]. Endovascular treatment preserving antegrade blood flow seems to be a reasonable treatment, but the long-term outcome remains unclear.

We report the case of a patient with a ruptured BVDA who was treated with a combined approach under bypass assistance, which resulted in a favorable outcome.

## Case presentation

**Figure 1:**
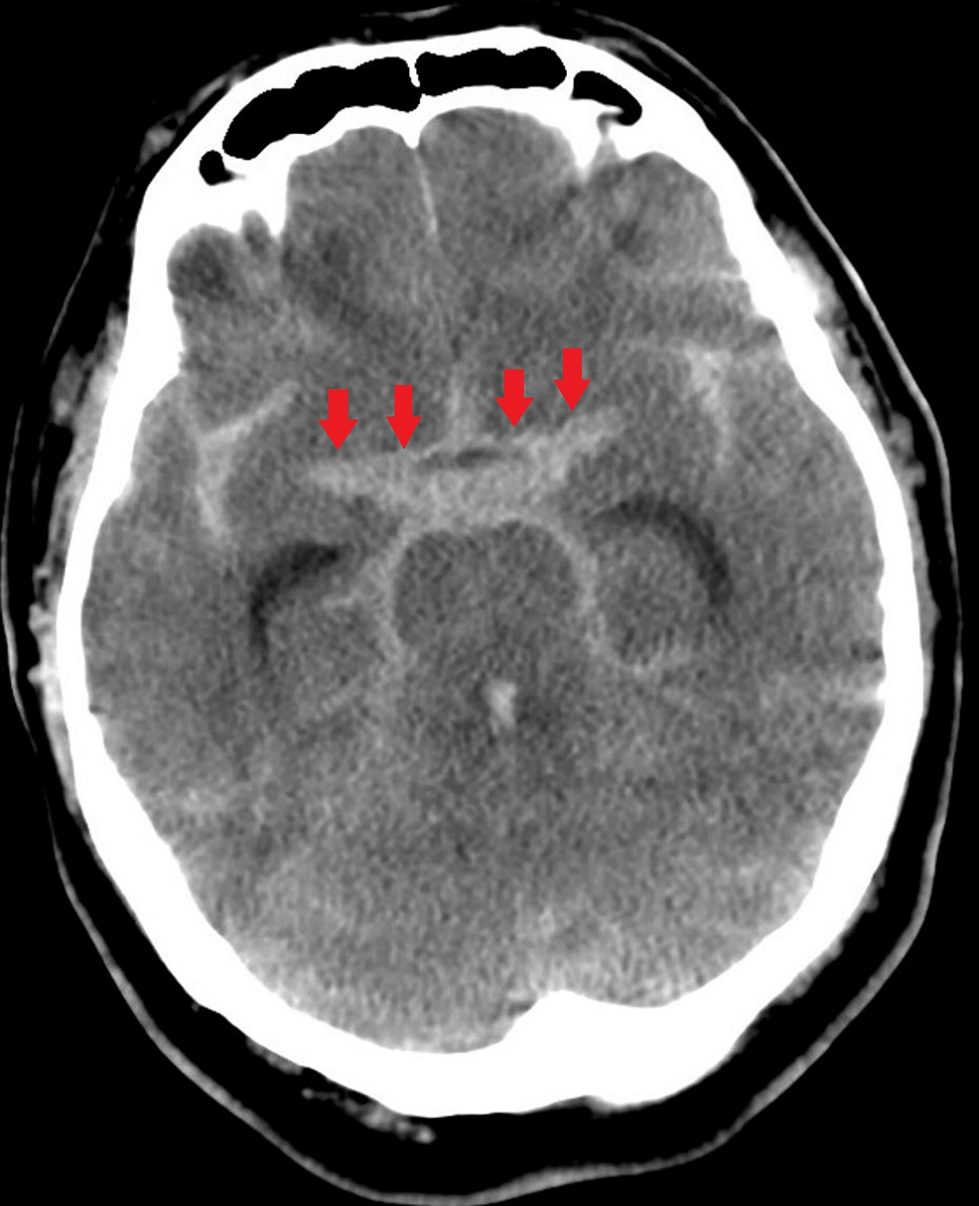
Head computed tomography Head computed tomography showed subarachnoid hemorrhage (Fisher’s classification 3)

A 57-year-old male was admitted to our hospital with a sudden onset of unconsciousness. His consciousness level was Glasgow Coma Scale (GCS) E1V1M4. His head computed tomography showed subarachnoid hemorrhage (Fisher’s classification 3) (Figure 1).

**Figure 2:**
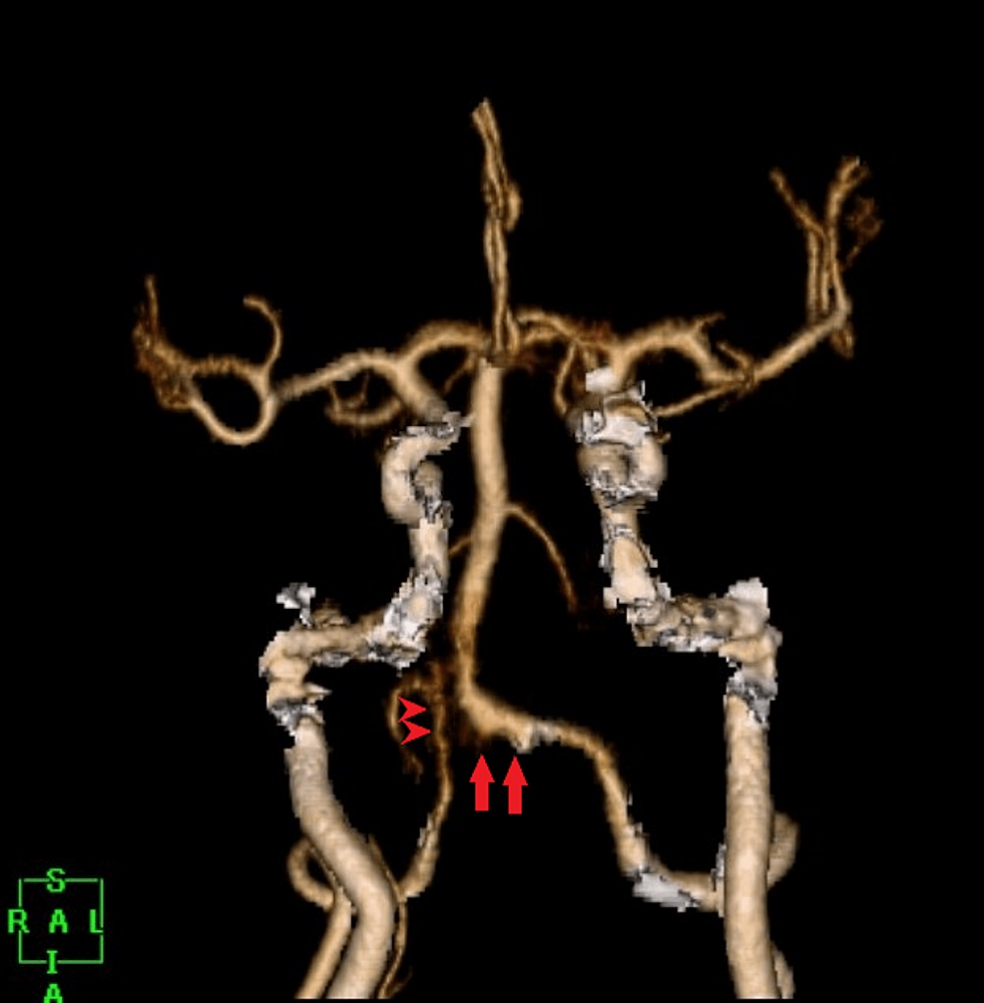
Preoperative 3DCTA 3DCTA showed an irregular, dilated surface wall of the left VA (arrows) and a narrowed right VA (arrowheads). The VA junction was high and deviated to the right side

Three-dimensional computed tomography angiography (3DCTA) showed an irregular and dilated surface wall of the left VA. Moreover, the right VA was narrowed to the VA junction. We suspected a BVDA (Figure 2). We considered that the ruptured point might be a dilated left VA, and the false lumen of the dissection might extend to the contralateral VA beyond the VA junction. We assumed that the extension of the false lumen in the right VA narrowed the true lumen. The left VA was dominant and much larger than the right VA.

**Figure 3:**
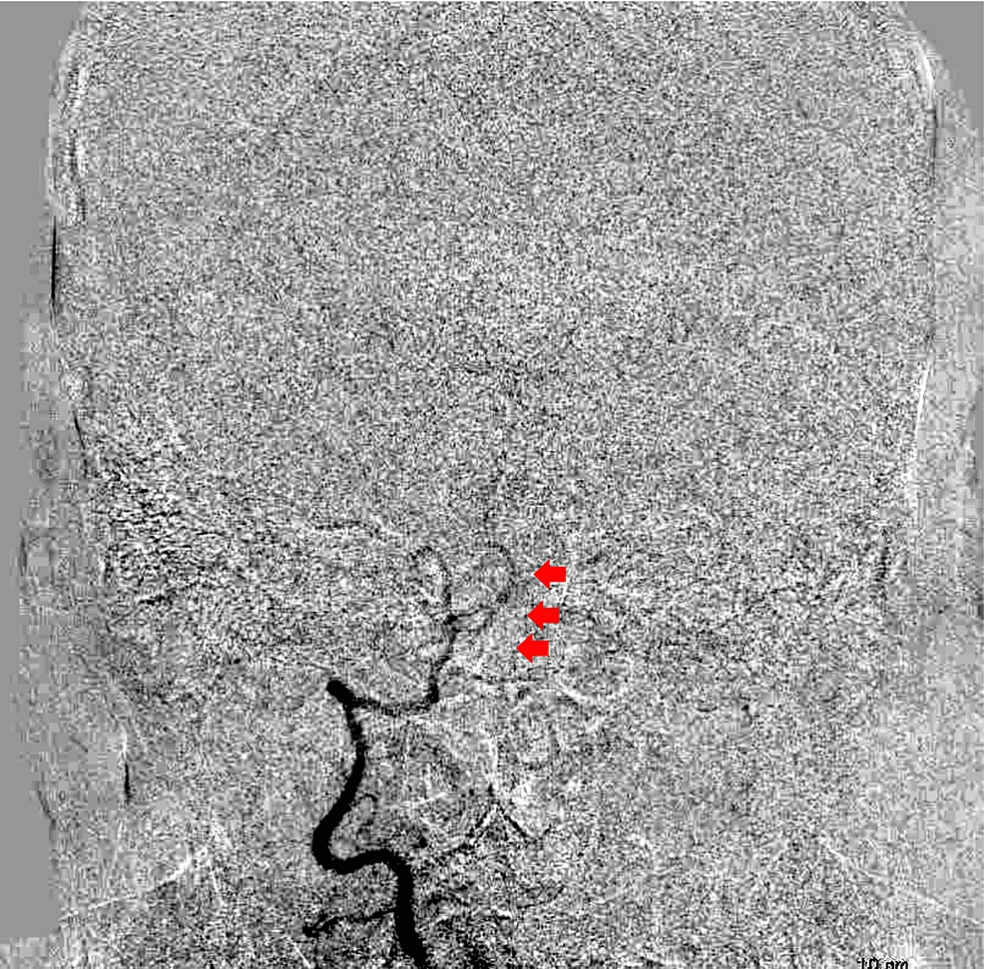
Preoperative digital subtraction angiography Digital subtraction angiography showed little blood flow through the right VA (arrows)

Digital subtraction angiography showed little blood flow through the right VA (Figure 3). We were unsure whether the right VA could supply sufficient blood flow to the brainstem when left vertebral dominant artery occlusion or trapping of the left VA dissection was performed.

Moreover, the VA junction was significantly deviated and high, such that distal end clipping of the left VA dissection was perceived to be difficult. The patient’s consciousness level was so bad that a balloon occlusion test (BOT) of the left VA was not applicable.

We planned the following operation: First, we performed a right superficial temporal artery-superior cerebellar artery (STA-SCA) bypass to secure the blood flow to the brainstem after proximal occlusion of the dominant left VA. Then, we performed proximal clipping of the left VA with the assistance of the STA-SCA bypass.

Operation

Under general anesthesia, at the park bench position, we performed a right STA-SCA bypass surgery. Prior to the STA-SCA bypass, we performed right ventricular drainage to evacuate cerebrospinal fluid. A linear skin incision was made along the right STA parietal branch, and the graft was harvested. Then, a curved skin incision meeting the prior linear skin incision, which formed an “h” shape skin incision, was performed. The right STA-SCA bypass was performed via the right subtemporal approach. The indocyanine green test showed good bypass patency. After completion of the STA-SCA bypass, we performed left VA proximal clipping in the prone position and suboccipital approach, without any complications.

**Figure 4:**
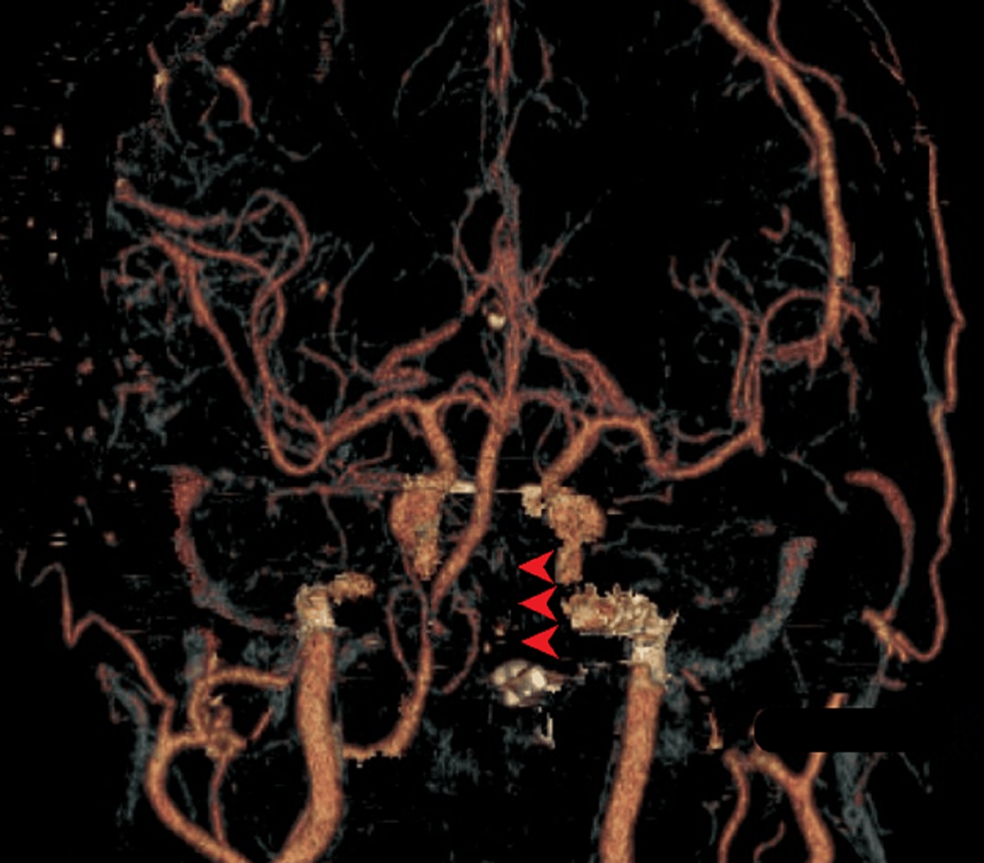
Postoperative 3DCTA 3DCTA showed complete obliteration of the left VA (arrowheads), but STA-SCA bypass was not clearly seen

The patient’s consciousness improved shortly after the operation (GCS=E4VTM6). However, with his history of radiation therapy for right parotid gland cancer and laryngomalacia, immediately after extubation, the patient experienced airway restriction. Therefore, extubation and tracheostomy were performed; 3DCTA on postoperative day (POD) 3 showed left VA obliteration (Figure 4). Although STA was palpable, a bypass was not clearly shown owing to the low resolution of CTA.

**Figure 5:**
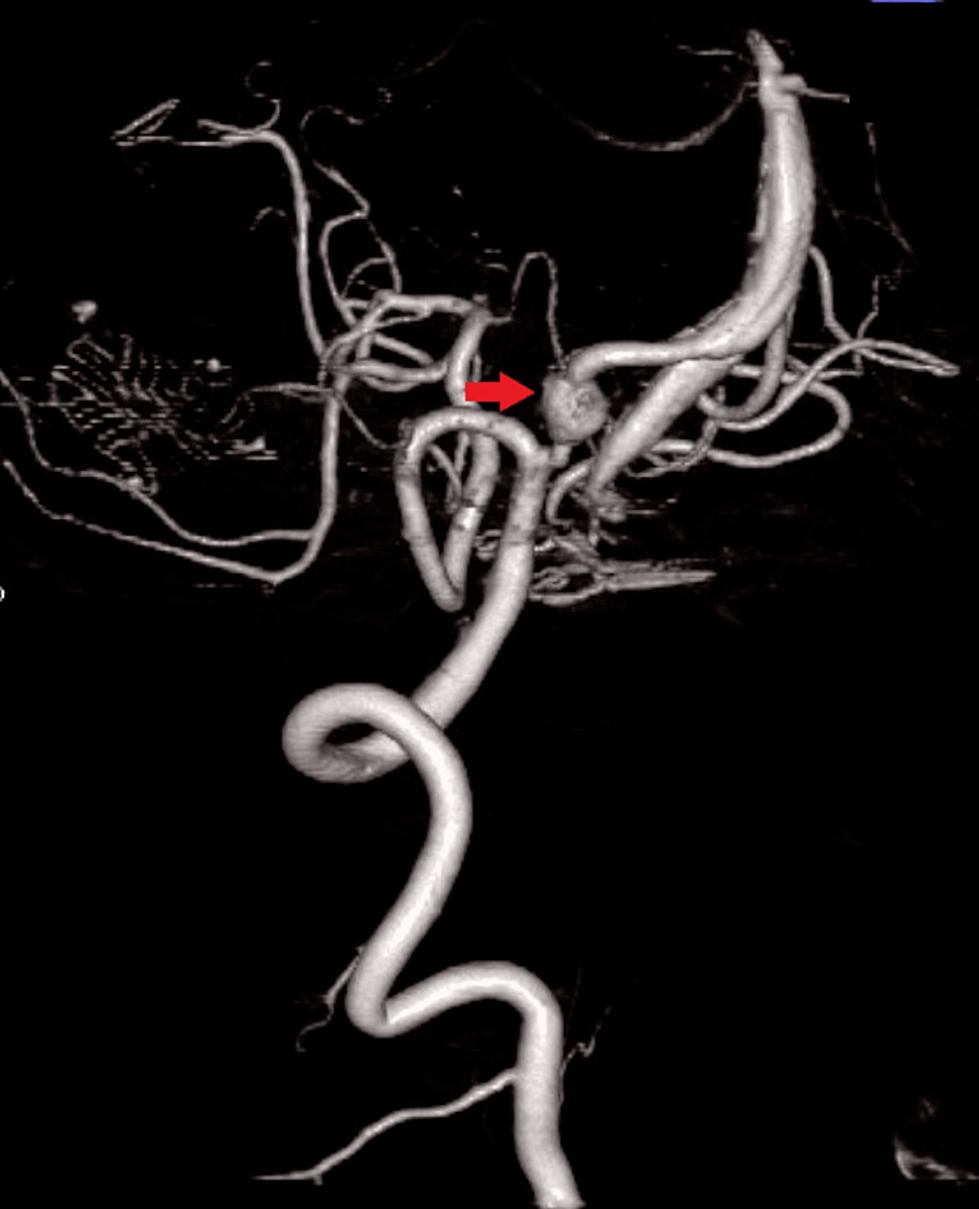
Follow-up 3DDSA 3DDSA right anterior oblique view. The fusiform aneurysm emerged on the left VA (arrow)

Palpation of the STA-SCA bypass disappeared on POD6, but there were no additional neurological symptoms. However, three-dimensional digital subtraction angiography (3DDSA) (POD15) showed the emergence of a fusiform aneurysm with a diameter of approximately 6 mm in the right VA (Figure 5).

The left VA was already occluded, and the right STA-SCA bypass was also thrombosed; therefore, the antegrade blood flow of the right VA had to be preserved. Stent-assisted coil embolization in the fusiform aneurysm appeared to be a potential therapy; however, the right proximal VA, just below the fusiform aneurysm, was stenosed with a diameter of 1.5 mm. This VA stenosis could have caused the catheter to wedge into the VA resulting in brainstem ischemia during the stenting procedure. We finally decided to perform stent-assisted coil embolization in the right VA following the left STA-SCA bypass to secure blood flow to the brainstem during the stenting procedure.

**Figure 6:**
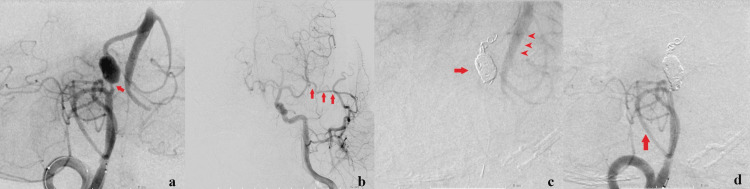
Preoperative and postoperative angiography (a) Preoperative angiography. The progression of right VA proximal stenosis was seen (arrow). (b) The STA-PCA bypass is patent (arrows). (c) Postoperative left carotid angiography. The internal trapping was completed (arrow). Retrograde blood flow to the basilar artery and distal VA was observed (arrowheads). (d) Postoperative right VA angiography. Complete obliteration of the fusiform aneurysm and preservation of the right PICA (arrow) were performed

On day 31, we attempted to perform a left STA-SCA bypass in the same fashion as the first operation. The left SCA deviated laterally and could not be found; however, we could find the posterior cerebral artery (PCA) and, thus, performed an STA-PCA bypass in the ambient cistern without any trouble. The total clamp time of PCA was 38 minutes. Two days after the bypass surgery, stent-assisted coil embolization was planned by an endovascular surgeon. A 5 Fr catheter was guided and placed in the right VA via the right femoral artery, and a 4 Fr diagnostic catheter was guided to the left common carotid artery via the left femoral artery. Preoperative angiography showed progression of the right proximal VA stenosis (Figure 6a). The diameter of the stenotic lesion was approximately 1 mm. Because implantation of a microstent in a 1-mm diameter vessel is usually off-label use and because of the potential risk of thrombotic complication and the risk of the increased difficulty of endovascular surgery, we considered changing the treatment strategy from the originally planned stent-assisted coil embolization. The left common carotid artery angiography showed excellent patency of the STA-PCA bypass (Figure 6b). The right VA angiography showed that the posterior inferior cerebellar artery (PICA) branched off just proximal to the aneurysm. We supposed that antegrade blood flow of the right VA would flow out to the PICA when internal trapping of the right VA was performed. We performed a right VA BOT for 10 minutes, but the somatosensory evoked potential (SEP) did not change. Moreover, angiography showed sufficient retrograde blood flow to the posterior circulation during the right VA BOT. Based on the BOT results, we finally decided to perform internal trapping of the right VA fusiform aneurysm, expecting sufficient retrograde blood flow from the STA-PCA bypass. Internal trapping of the fusiform aneurysm was completed without complications. Postoperative angiography showed retrograde blood flow to the basilar artery and distal VA (Figure 6c) and preservation of the PICA (Figure 6d).

**Figure 7:**
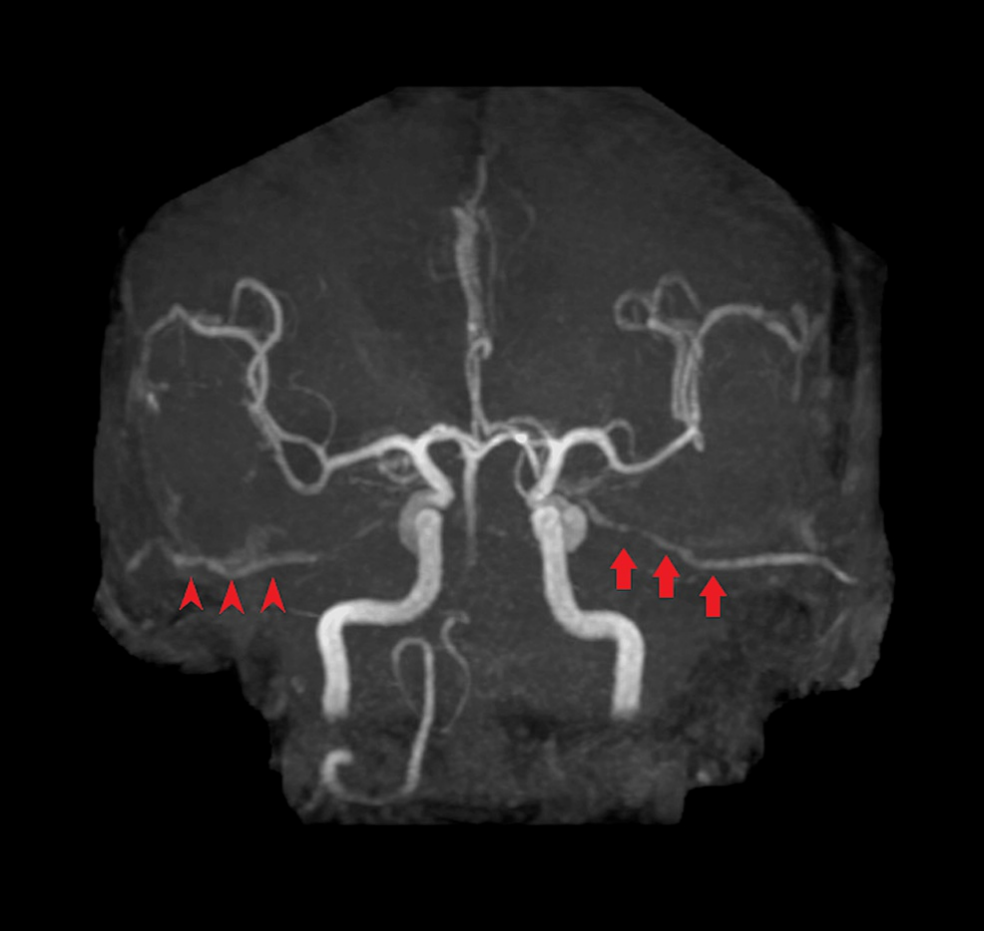
Postoperative MRA Follow-up MRA showed left STA-PCA patency (arrows), right STA-SCA bypass recanalization (arrow), and BVDA obliteration. The basilar artery was perfused with retrograde blood flow from the bypass

SEP did not change during the procedure, and the patient’s postoperative course was uneventful. The magnetic resonance angiography (MRA) showed excellent patency of the left STA-PCA bypass, recanalization of the right STA-SCA bypass, and complete obliteration of the BVDA (Figure 7). There was no additional neurological deficit after endovascular treatment. The patient was transferred to a rehabilitation hospital (mRS=2, GCS=E4VTM6).

## Discussion

BVDA is a clinically challenging disease, and its optimal treatment has not yet been established. A treatment strategy for BVDA was reported [5]. Similar to our case, it was reported that occlusion of the unilateral VA caused enlargement of the contralateral VA aneurysm due to increased hemodynamic stress on the contralateral side [5]. Trapping with a V3-V3 or V3-V4 bypass was recommended [5]. Reconstruction of VA blood flow facilitates trapping of the contralateral side when the contralateral VA dissection enlarges. In our case, the union of the VA was so high and deviated that we anticipated that trapping and V3-V4 or V3-V3 bypass operation would be very difficult. In addition, an endovascular surgeon was unavailable in our institution during the first operation, and internal trapping of the VA was not feasible. Furthermore, stenting in the acute phase of subarachnoid hemorrhage is considered an off-label use under the Japanese health insurance system. Therefore, we decided to perform an STA-SCA insurance bypass prior to the proximal occlusion of the left VA during the first operation. At first, the left VA seemed to be thrombosed without any ischemic complication following proximal clipping of the left VA. However, a contralateral VA aneurysm appeared two weeks after the operation with the thrombosis of the right STA-SCA bypass. We suppose that increased hemodynamic stress of antegrade blood flow on the right VA might cause the emergence of the aneurysm and delayed thrombosis of the right STA-SCA bypass. Finally, we performed internal trapping of the right VA with the assistance of a left STA-PCA bypass, without any complications. Compared with high-flow bypass, our strategy was less invasive and yielded good results for the patient. High-flow bypass, such as V3-radial artery graft-P2a bypass, could supply more sufficient blood flow than low-flow bypass. If a high-flow bypass strategy had been chosen during the first operation, we could simply have made internal trapping of the right VA without the assistance of the left STA-PCA insurance bypass. High-flow bypass using an interposition graft is a more invasive surgery than in situ graft bypass, such as STA-SCA bypass, but could reduce additional insurance bypass surgery.

After the emergence of a fusiform aneurysm on the contralateral VA in the subacute phase, our strategy involved stent-assisted coil embolization in the aneurysm to preserve antegrade blood flow in the contralateral vertebral and basilar arteries [6,9]. However, the contralateral VA was so stenosed that we had to change our strategy to internal trapping of the aneurysm. Fortunately, our STA-PCA insurance bypass was perfectly patent and supplied sufficient blood flow, but there was some risk that the STA-PCA low-flow bypass did not supply sufficient blood flow to the brainstem. We performed BOT of the right VA just before the internal trapping of the right VA, but BOT should be performed before making a stenting strategy. On the basis of the preoperative BOT result, we might have been able to choose the appropriate high-flow or low-flow bypass procedure [8,10].

BVDA is a challenging and lethal disease, and its treatment remains controversial. There is no typical treatment for BVDA, and the surgical strategy should be thoroughly discussed based on individual anatomy and patient condition. In our case, we applied multimodal treatment with vascular reconstruction to the posterior circulation and had a favorable outcome. The key to controlling this challenging aneurysm is direct vascular reconstruction of the posterior circulation, which requires an advanced bypass technique.

## Conclusions

BVDA is a rare and challenging disease. The treatment strategy must be thoroughly discussed for each case. Proximal artery occlusion or trapping with insurance bypass of the posterior circulation are potentially lifesaving treatments, although unilateral VA occlusion may cause enlargement of the contralateral VA dissecting aneurysm with hemodynamic stress. Multimodal treatment is a potential treatment option for the emerging contralateral dissecting aneurysm.
